# GSK3β/HIF-1α signaling-dependent anti-parasite effect of *Cynanchi atrati Radix*

**DOI:** 10.1016/j.isci.2025.114292

**Published:** 2025-12-01

**Authors:** Fei-Fei Gao, Guan-Hao Hong, Xin-Cheng Wang, Jia-hui Zeng, Yu-Sun Yun, In-Wook Choi, Jae-Min Yuk, Wei Zhou, Xin-tian Chen, Gang Min Hur, Guang-Ho Cha

**Affiliations:** 1 Stem Cell Research and Cellular Therapy Center, Affiliated Hospital of Guangdong Medical University, Zhanjiang 524001, Guangdong, China; 2 Laboratory of Obstetrics and Gynecology, Department of Obstetrics and Gynecology, Affiliated Hospital of Guangdong Medical University, Zhanjiang 524001, Guangdong, China; 3 Guangdong Provincial Key Laboratory of Autophagy and Major Chronic Non-communicable Diseases, Zhanjiang 524001, Guangdong, China; 4 Brain Korea 21 FOUR Project for Medical Science, Chungnam National University College of Medicine, Daejeon 301-131, Korea; 5 Department of Medical Science and Department of Infection Biology, Chungnam National University, College of Medicine, Daejeon 301-131, Korea; 6 Biotissue Repository, Affiliated Hospital of Guangdong Medical University, Zhanjiang 524001, Guangdong, China; 7 Department of Gastroenterology, Affiliated Hospital of Guangdong Medical University, Zhanjiang 524001, Guangdong, China; 8 Department of Pharmacology, Research Institute for Medical Science, College of Medicine, Chungnam National University, Daejeon 35015, Republic of Korea

**Keywords:** parasitology, microbial metabolism, microbiology parasite

## Abstract

Medicinal plants yield bioactive compounds with potential for parasite control. We examined *Cynanchi atrati Radix* (*C. atrati*) and its component 4′-hydroxyacetophenone (4′HAP) for activity against *Toxoplasma gondii* (*T. gondii*) using cultured cells and mouse infection models. *C. atrati* extracts limited parasite growth with minimal host-cell toxicity. Chemical screening pinpointed 4′HAP as the active constituent that suppresses *T. gondii* proliferation *in vitro* and *in vivo*. Mechanistically, *C. atrati* and 4′HAP activated GSK3β, destabilized HIF-1α, and curtailed parasite fitness; pharmacologic GSK3β inhibition restored parasite growth, whereas HIF-1α depletion further reduced survival, highlighting the GSK3β/HIF-1α axis as a host pathway that constrains infection. These results identify a plant-derived small molecule and its mechanistic target for host-directed antiparasitic therapy and provide a framework for developing treatments for toxoplasmosis.

## Highlights

• *C. atrati* and its bioactive compound 4′HAP can suppress *T. gondii* proliferation
• *C. atrati* and 4′HAP inhibit host GSK3β/HIF-1α signaling by reducing HIF-1α stability
• GSK3β activation is essential for the anti-parasitic effects of *C. atrati* and 4′HAP
• *C. atrati* or 4′HAP are promising anti-parasitic candidates for ocular toxoplasmosis

## Graphical abstract

**Figure:**
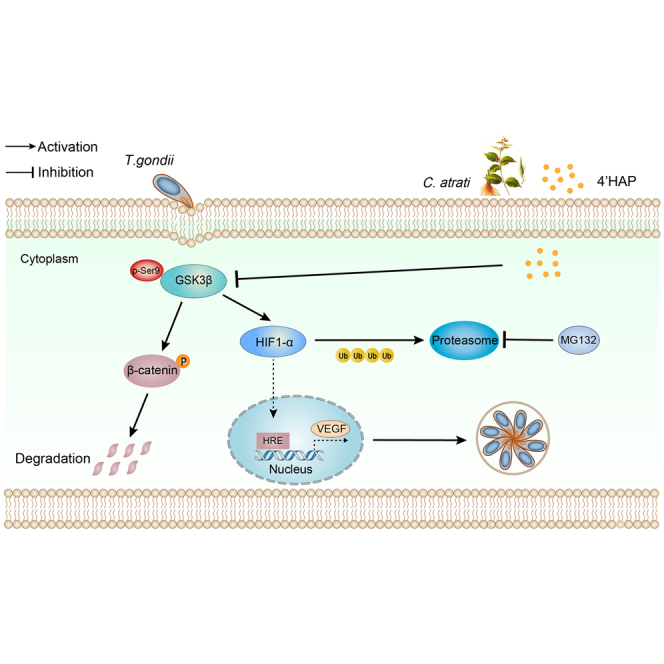

## Introduction

*Toxoplasma gondii* (*T. gondii*) is a widespread intracellular protozoan parasite that can infect almost all warm-blooded animals, including humans. Although infections are usually asymptomatic in immunocompetent individuals, they can cause severe complications in immunocompromised patients and congenital cases, including encephalitis, chorioretinitis, and systemic disease.^1^^,^^2^ *T. gondii* manipulates host cellular pathways to ensure its survival and replication, highlighting its pathogenic success and emphasizing the need to study host-parasite interactions to develop novel therapeutic strategies.^3^

*Cynanchum atratum Bunge* (Baiwei), pharmacologically recognized as *Cynanchi atrati Radix* (*C. atrati*) in the Chinese pharmacopoeia,^4^ is a traditional east Asian medicinal herb with proven anti-tubercular,^5^ antiviral,^6^ and antitumor properties.^7^ Emerging evidence suggests that *C. atrati* exhibits anti-inflammatory potential by modulating macrophage responses in Raw264.7 and N9 microglial cells,^8^^,^^9^ primarily through the suppression of IKK/NF-κB signaling, which subsequently inhibits proinflammatory mediators such as iNOS and COX-2.^10^ Phytochemical analyses have identified over 100 bioactive compounds in *C. atrati*, including unique C21 steroidal glycosides,^11^ pregnane derivatives,^12^ seco-pregnane steroidal glycoside alkaloids,^6^ and phenolics such as 2,4-dihydroxyacetophenone.^13^ Notably, cynatratoside-C, a significant component of *C. atrati*, has demonstrated anti-parasitic activity against *Ichthyophthirius multifiliis*, indicating that certain constituents of this herb may possess anti-protozoal properties. However, its potential efficacy against intracellular parasites, particularly *T. gondii*, remains unexplored.

Our team previously demonstrated that *T. gondii* infection significantly upregulates VEGF expression at both transcriptional and translational levels in ARPE-19 cells, strictly depending on parasite burden and infection duration.^14^ Our investigations revealed the simultaneous upregulation of hypoxia-inducible factor 1-alpha (HIF-1α), the key transcriptional regulator of VEGF and cellular hypoxia responses, indicating a coordinated regulatory mechanism.^14^ Notably, chronic cerebral *T. gondii* infection preconditions neural tissue by elevating baseline HIF-1α expression before middle cerebral artery occlusion, suggesting a potential therapeutic role in ischemic neuroprotection via HIF-1α-mediated adaptive responses.^15^

The parasite’s ability to stabilize HIF-1α under normoxic conditions presents a compelling mechanistic puzzle. Current evidence suggests that *T. gondii* disrupts oxygen-dependent degradation pathways by inhibiting the prolyl hydroxylation of HIF-1α, thereby preventing its proteasomal degradation.^16^ Central to this regulatory network is glycogen synthase kinase 3 beta (GSK3β), a multifunctional kinase that normally promotes HIF-1α degradation.^17^^,^^18^^,^^19^ Intriguingly, *T. gondii* infection seems to modulate GSK3β activity differently depending on parasite genotype and developmental stage. While the Wh6 strain of the Chinese Ⅰ genotype induces tau phosphorylation via GSK3β activation,^20^ the parasite also employs sophisticated molecular strategies to exploit this kinase.

Parasite-derived effector proteins, particularly the dense granule protein GRA18, directly interact with host signaling components, forming ternary complexes with GSK3 and PP2A-B56 phosphatase to activate β-catenin signaling and reprogram host gene expression.^21^ Furthermore, *T. gondii* has evolved TgGSK, a functional GSK3 homolog that is essential for parasite cell cycle progression via centrosome regulation and endodyogeny.^22^ This dual exploitation of host- and parasite-encoded GSK3-like kinases suggests an evolutionarily conserved strategy for manipulating cellular pathways.

Comparative parasitological studies highlight the therapeutic relevance of GSK3β modulation. In *Leishmania* infections, pathogen-induced phosphorylation of GSK3β suppresses host defenses by modulating FOXO1-dependent inflammatory responses and apoptosis. Notably, genetic stabilization of GSK3β activity via constitutively active mutants markedly impairs parasite survival, supporting its potential as a therapeutic target.^23^

The complex parasite-host interface centered on GSK3β/HIF-1α cross-regulation offers new opportunities for targeted interventions. Strategically modulating this axis could both disrupt parasitic survival mechanisms and enhance host adaptive responses, providing a dual-pronged strategy against chronic infections and their pathological consequences.

This study systematically assesses the anti-parasitic efficacy of *C. atrati* against *T. gondii* infection in ARPE-19 cells and identifies 4′-Hydroxyacetophenone (4′HAP) as its main bioactive constituent. Mechanistic studies show that *C. atrati* extracts and purified 4′HAP exert strong anti-parasitic effects by selectively activating GSK3β, which in turn destabilizes HIF-1α under infection conditions. This dual modulation of the GSK3β/HIF-1α axis interferes with parasite-induced HIF-1α stabilization, a crucial survival strategy of *T. gondii*, thereby markedly inhibiting intracellular proliferation.

## Results

### *C. atrati* extract inhibits *T. gondii* proliferation in ARPE-19 cells

**Figure 1:**
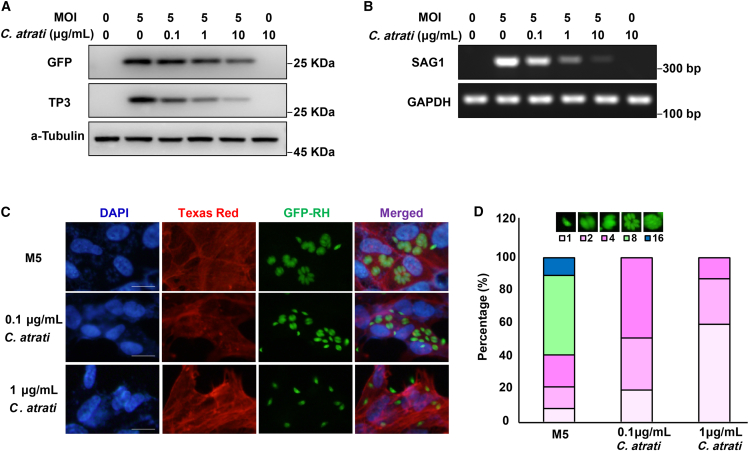
*C. atrati* suppresses the *T. gondii* growth (A) Western blot was used to detect the GFP/TP3 in ARPE-19 cells pretreated with *C. atrati* for 4 h at the indicated concentrations, followed by infection with GFP-RH at MOI5 (M5). (B) RT-PCR was used to detect the SAG1 mRNA levels in ARPE-19 cells pretreated with *C. atrati* for 4 h at the indicated concentrations, followed by infection with GFP-RH at MOI5. (C) ARPE-19 cells were infected with *T. gondii* at MOI5 and treated with *C. atrati* for 24 h, and then the *T. gondii* proliferation was measured by fluorescence microscopy. Scale bars, 20 μm. (D) The number of parasites per parasitophorous vacuole (PV) was counted and covered by percentage; a total of 100 parasitophorous vacuoles (PVs) were counted. Results are presented as the mean ± S.D. from three independent experiments.

To assess the anti-parasitic efficacy of *C. atrati* extract, we developed an *in vitro* model of ocular toxoplasmosis using ARPE-19 cells infected with green fluorescent protein (GFP)-expressing *T. gondii* at a multiplicity of infection (MOI) of five. Cells were exposed to *C. atrati* extract at varying concentrations (0.1, 1, and 10 μg/mL) for 24 h. Western blot analysis showed a dose-dependent decrease in GFP and the *T. gondii*-specific surface protein TP3 (SAG1/p30, also termed SRS29B; ToxoDB: TGME49_233460/TGGT1_233460) (Figure 1A). Concurrently, transcriptional profiling revealed significant downregulation of the *T. gondii* surface antigen gene SAG1 (Figure 1B), confirming that *C. atrati* extract suppresses parasite viability.

To further characterize replication inhibition, we quantified tachyzoite proliferation by counting the number of tachyzoites per PV (Figures 1C and 1D). Fluorescence microscopy revealed that after 24 h of infection, most *T. gondii* underwent three to four rounds of division, resulting in eight or more tachyzoites per parasitophorous vacuole (PV). However, pre-treatment with *C. atrati* extract (0.1 μg/mL) significantly reduced tachyzoite division. At a higher concentration (1 μg/mL) more than 50% of infected *T. gondii* failed to divide. To benchmark against standard-of-care and to generalize across cell types, we included pyrimethamine as a positive control and repeated the assays in HFF-1 fibroblasts. In ARPE-19 cells, immunofluorescence imaging and flow cytometry showed that *C. atrati* extract markedly reduced intracellular parasite burden, yielding GFP-positive fractions of ∼14.6% compared with ∼61% in the M5 medium control and slightly outperforming pyrimethamine at an equivalent test concentration (Figures S1A and S1B). Consistent effects were observed in HFF-1 cells, where *C. atrati* similarly shifted vacuole composition toward low-replication categories and decreased GFP-positive events to levels comparable to or below those achieved by pyrimethamine (Figures S2A and S2B). At the same time, the tested concentrations of *C. atrati* extract did not affect ARPE-19 cell growth or viability (Figure S3A).

These findings demonstrate that *C. atrati* extract exhibits anti-*T. gondii* activity by inhibiting parasite proliferation, and this effect is achieved at remarkably low concentrations without detectable host cytotoxicity. Furthermore, the dose-dependent bioactivity profile strongly suggests that *C. atrati* extract contains specific anti-protozoal constituents, likely small-molecule inhibitors targeting essential *T. gondii* proliferation pathways. These compounds could be valuable for developing next-generation protozoacidal agents with dual advantages: targeted action and host compatibility.

### Screening of *C. atrati* compounds for inhibiting *T. gondii* proliferation

**Figure 2:**
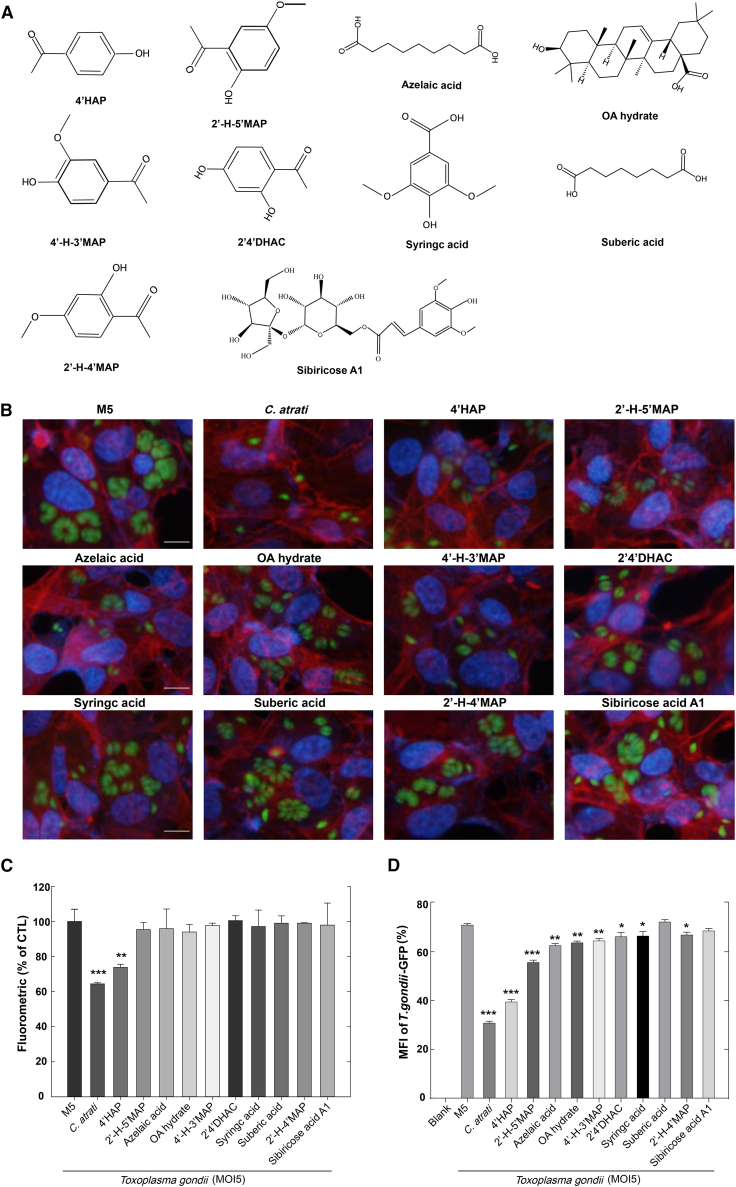
Screening of commercially sourced reference compounds reported from *C. atrati* for inhibition of *T. gondii* proliferation (A) The chemical structures of the *C. atrati-*derived compounds. (B) ARPE-19 cells were infected with *T. gondii* at MOI5 and treated with commercially sourced reference compounds reported from *C. atrati* (1 μg/mL) for 24 h. *T. gondii* proliferation was measured by fluorescence microscopy. Scale bars, 10 μm. (C) ARPE-19 cells were infected with *T. gondii* (MOI5) and treated with different commercially sourced reference compounds reported from *C. atrati* for 24 h. Fluoroskan Ascent FL was used to detect the fluorometric. Results are presented as the mean ± S.D. from three independent experiments. ∗∗, *p* < 0.01, ∗∗∗, *p* < 0.001 by Student’s *t* test. (D) ARPE-19 cells were pretreated with different commercially sourced reference compounds reported from *C. atrati* for 4 h and infected with *T. gondii* (MOI5) for 24 h. Median fluorescence intensity (MFI) was detected by FACS. Results are presented as the mean ± S.D. from three independent experiments. ∗, *p* < 0.05, ∗∗, *p* < 0.01, ∗∗∗, *p* < 0.001 by Student’s *t* test.

*C. atrati* extract consists of a mixture of bioactive molecules, and isolating a single active compound could yield a promising candidate for anti-*T. gondii* drug development. To identify the bioactive compound responsible for the anti-*T. gondii* activity of *C. atrati*, we screened individual, commercially sourced reference compounds reported from *C. atrati* (Figure 2A) and evaluated their effects on *T. gondii* growth.

Using the GFP-expressing *T. gondii* RH strain, we quantified *T. gondii* proliferation in host cells to evaluate the impact of each molecule on parasite growth and replication. Immunocytochemistry-based microscopic screening for *T. gondii* proliferation inhibitors showed that among the tested compounds, 4′HAP exhibited the strongest inhibitory effect on *T. gondii* proliferation compared to other candidates (Figure 2B). Fluorometric-based screening of *C. atrati* compounds for *T. gondii* growth inhibition revealed that among the ten tested constituents, 4-Hydroxyacetophenone (4′HAP) exhibited an anti-*T. gondii* efficacy comparable to that of *C. atrati* extract (Figure 2C). Consistently, FACS-based screening also showed that 4′HAP exhibited an inhibitory effect on *T. gondii* growth similar to that of *C. atrati* extract (Figures 2D and S4).

### 4′HAP remarkably inhibited *T. gondii* growth in ARPE-19 cells

**Figure 3:**
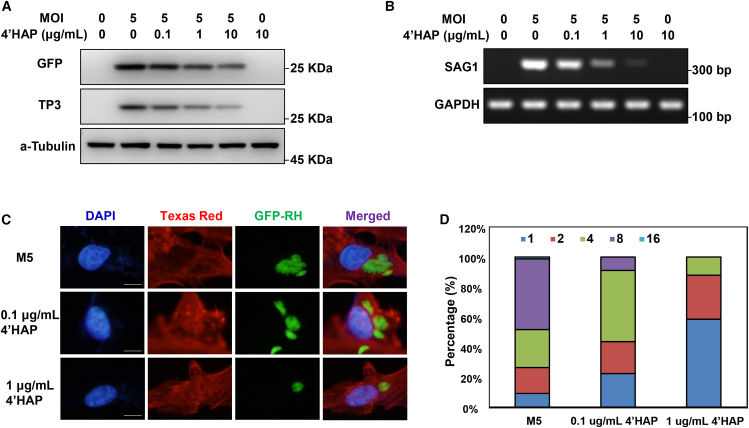
4′HAP suppresses the *T. gondii* growth (A) Western blot was used to detect the GFP/TP3 proteins in ARPE-19 cells pretreated with 4′HAP for 4 h at the indicated concentrations, followed by infection with GFP-RH at MOI5. (B) RT-PCR was used to detect the SAG1 mRNA. (C) ARPE-19 cells were infected with *T. gondii* at MOI5 and treated with 4′HAP for 24 h, and then the *T. gondii* proliferation was measured by fluorescence microscopy, 5 μm. (D) The number of parasites per PV was counted and covered by percentage; a total of 100 PVs were counted. Results are presented as the mean ± S.D. from three independent experiments.

Based on the initial screening, we selected 4′HAP for further analysis and re-evaluated its inhibitory effects on *T. gondii* growth. 4′HAP significantly reduced GFP and TP3 protein levels, as well as SAG1 mRNA levels, in a dose-dependent manner (Figures 3A and 3B). We also assessed the efficacy of 4′HAP in inhibiting *T. gondii* replication through direct microscopic observation. As shown in Figure 3C, treatment with 0.1 μg/mL of 4′HAP significantly suppressed *T. gondii* replication, while 1 μg/mL led to an even stronger inhibitory effect. Quantitative analysis of PVs showed that, in the control group, most PVs contained eight tachyzoites, indicating active replication. In contrast, treatment with 0.1 μg/mL 4′HAP shifted the distribution, with PVs containing four tachyzoites becoming predominant. At 1 μg/mL, more than 50% of PVs contained a single tachyzoite, suggesting that 4′HAP strongly inhibits *T. gondii* cell division (Figure 3D). At the same time, the tested concentrations of 4′HAP did not affect ARPE-19 cell growth or viability (Figure S1B).

### Integrative target prediction and enrichment analysis to identify the GSK3β/HIF-1α axis in 4′HAP-mediated anti-*T. gondii* activity

**Figure 4:**
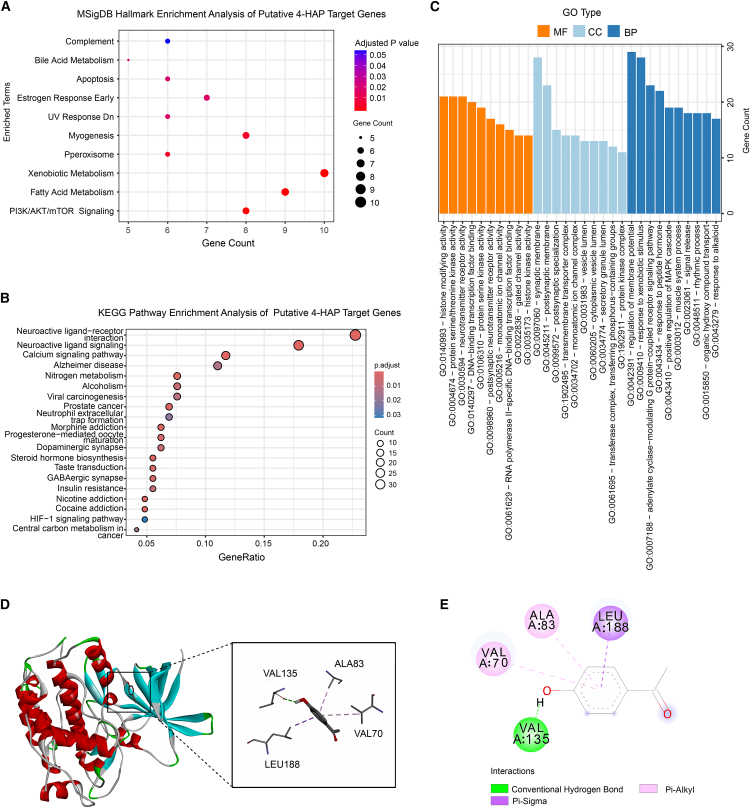
Network pharmacology and molecular docking identify GSK3β as a key regulator of the HIF-1α signaling pathway in 4′HAP-mediated anti-*Toxoplasma gondii* activity (A) Bubble chart showing MSigDB Hallmark enrichment analysis of putative 4′HAP target genes. The top enriched Hallmark gene sets are displayed with corresponding adjusted *p*-values. Dot color represents the adjusted *p*-value, while dot size corresponds to the number of genes associated with each term. (B) Bubble chart of KEGG pathways analysis. Top 20 pathways with corresponding *p*-values, displayed in a dot plot. The color scales indicated *p*-values and the sizes of the dots represented the gene count of each term. (C) GO enrichment analysis. The length and color of the bands were used to characterize the number of targets involved into the related biological processes. (D) 3D model of GSK3β (PDBID:1J1B) crystal structure docking. (E) GSK3β docking 2D model.

To investigate the potential mechanism of 4′HAP against *T. gondii*, a total of 194 potential targets of 4′HAP were initially screened using the TCMSP, SwissTargetPrediction, Charité Prediction platform, and PubMed databases. After removal of duplicate entries, 188 unique candidate targets were identified. MSigDB Hallmark enrichment analysis demonstrated that these targets were significantly associated with multiple biological pathways (Figure 4A). Among them, the PI3K/AKT/mTOR signaling pathway was the most^18^ significantly enriched, indicating it as a potential key regulatory axis of 4′HAP. Further KEGG pathway enrichment analysis revealed that the targets were involved in diverse signaling cascades, including neuroactive ligand-receptor interaction, calcium signaling, and notably, the HIF-1 signaling pathway (Figure 4B). The identification of HIF-1 signaling is particularly relevant, because it has been reported that during *T. gondii* infection, HIF-1α and hexokinase 2 collaborate to reprogram host cell metabolism under normoxic conditions, creating a favorable environment for parasite proliferation.^24^To further characterize the functional profile of these targets, gene ontology enrichment analysis was performed (Figure 4C). Remarkably, serine/threonine kinase activity emerged as one of the top-ranked molecular functions, underscoring the importance of kinase-related regulation in the proposed mechanism of action. Given the strong association of serine/threonine kinase activity and our previous findings implicating inhibition of glycogen synthase kinase 3 beta (GSK3β) as an essential regulator of *T. gondii* proliferation, we selected GSK3β as a representative candidate target for further validation. To explore the potential interaction between 4′HAP and GSK3β, molecular docking simulations were conducted using AutoDock Vina to assess the binding of 4′HAP within the ATP-binding pocket of GSK3β. The results demonstrated a stable binding conformation of 4′HAP within the active site of GSK3β, characterized by hydrophobic interactions with VAL70, ALA83, VAL135, and LEU188 residues, and a conventional hydrogen bond formed with VAL135 (Figures 4D and 4E). Based on these combined findings, we hypothesized that the GSK3β/HIF-1α axis may be critically involved in mediating the anti-parasitic effects of 4′HAP against *T. gondii*.

### *C. atrati* inhibits *T. gondii* proliferation through destabilization of HIF-1α

**Figure 5:**
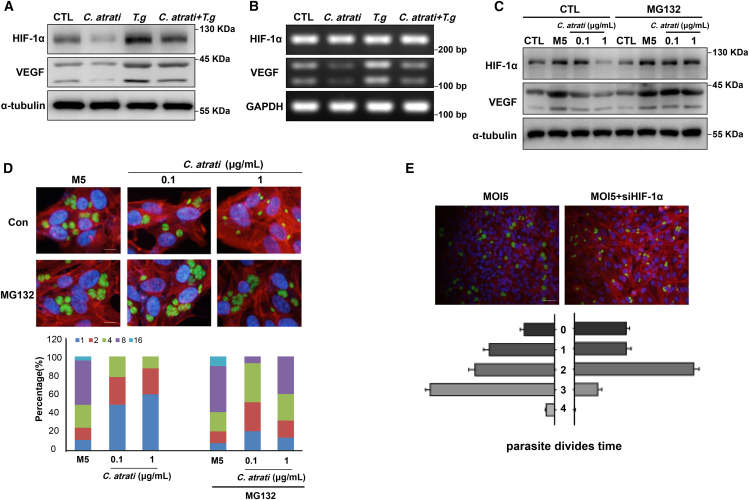
*C. atrati* represses *T. gondii* growth by reducing HIF-1α stability. ARPE-19 cells were pretreated with *C. atrati* (1 μg/mL) for 4 h and infected with *T. gondii* (MOI5) for 24h or left uninfected (CTL) Western blot (A) and RT-PCR (B) were performed to detect the protein and mRNA levels of HIF-1α and VEGF, respectively. ARPE-19 cells were pretreated with MG-132 or vehicle control for 4 h, treated with *C. atrati* for 4 h at the indicated concentrations*,* and then infected with *T. gondii* for 24 h. (C) The protein levels of HIF-1α and VEGF were measured by Western blot. (D) *T. gondii* proliferation was measured by fluorescence microscopy. Scale bars, 10 μm. The number of parasites per PV was counted and covered by percentage; a total of 100 PVs were counted. Results are presented as the mean ± S.D. from three independent experiments. (E) ARPE-19 cells were transiently transfected with HIF-1α siRNA (siHIF-1α) and then infected with *T. gondii* (MOI5); the parasite division times were analyzed by fluorescence microscopy; a total of 100 PVs were counted. Scale bars, 50 μm. Results are presented as the mean ± S.D. from three independent experiments.

To confirm the GSK3β/HIF-1α is important for 4′HAP against *T. gondii*. Therefore, we examined the effects of *C. atrati* on HIF-1α activity. The results showed that *C. atrati* extract attenuated *T. gondii*-induced upregulation of HIF-1α protein levels (Figure 5A), while mRNA expression levels remained unchanged (Figure 5B). This suggests that *C. atrati* regulates *T. gondii*-mediated HIF-1α at the protein stability level rather than through transcriptional changes. To validate this hypothesis and explore the underlying mechanism, we treated cells with the proteasome inhibitor MG-132 and found that MG-132 reversed the suppressive effect of *C. atrati* extract on *T. gondii*-induced HIF-1α upregulation (Figure 5C). These findings indicate that *C. atrati* regulates HIF-1α activity by modulating its protein stability. Moreover, the levels of vascular endothelial growth factor (VEGF), a well-known downstream target of HIF-1α, were significantly increased at both transcriptional and translational levels (Figures 5A–5C). However, pre-treatment with *C. atrati* suppressed *T. gondii*-induced VEGF expression, suggesting that *C. atrati* modulates host-pathogen interactions through HIF-1α regulation.

In addition, MG-132 treatment significantly reversed the suppression of *T. gondii* growth induced by *C. atrati* extract. Specifically, MG-132 supplementation markedly increased the proportion of PVs containing eight tachyzoites (Figure 5D), suggesting that *C. atrati* inhibits *T. gondii* replication by destabilizing HIF-1α protein. Based on these findings, we hypothesized that HIF-1α stability is crucial for *T. gondii* proliferation. To verify this hypothesis, we examined *T. gondii* proliferation in HIF-1α-silenced host cells using fluorescence microscopy (Figure 5E). As a result, in the absence of HIF-1α, *T. gondii* replication was severely impaired, with a significant reduction in the proportion of parasites undergoing three rounds of division.

### *C. atrati* blocks *T. gondii* growth via GSK3β-mediated HIF-1α destabilization

**Figure 6:**
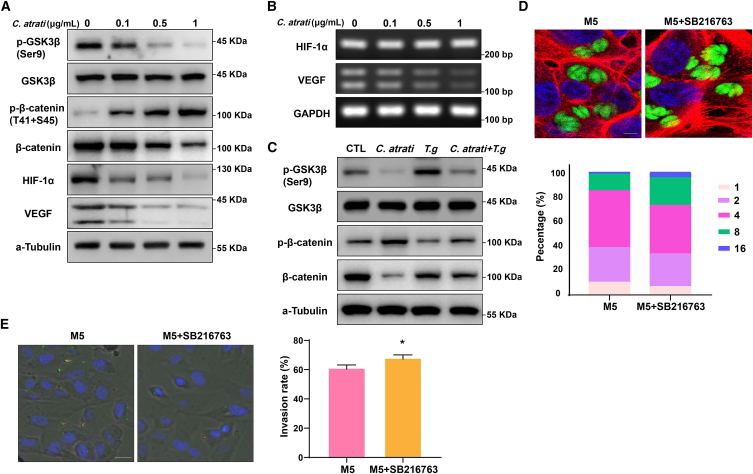
*C. atrati* reduces HIF-1α stability by GSK3β/β-catenin pathway (A) Western blot analysis of phosphor- GSK3β/β-catenin and total- GSK3β/β-catenin, as well as HIF-1α and VEGF in ARPE-19 cells treated with *C. atrati* for 24 h. (B) mRNA levels of HIF-1α and VEGF were measured by RT-PCR. (C). ARPE-19 cells were pretreated with *C. atrati* (1 μg/mL) and infected with *T. gondii* (MOI5) or left uninfected (CTL), and then the protein levels of phosphor-GSK3β/β-catenin and total-GSK3β/β-catenin were analyzed. (D) ARPE-19 cells were pretreated with GSK3β inhibitor (SB216763) for 4 h and then infected with *T. gondii* (MOI5) for 24 h. The samples were imaged by confocal microscopy. The number of parasites per PV was counted and covered by percentage; a total of 100 PVs were counted. Scale bars, 5 μm. Results are presented as the mean ± S.D. from three independent experiments. (E) ARPE-19 cells were pretreated with GSK3β inhibitor (SB216763) for 4 h and then infected with *T. gondii* (MOI5) for 24 h. The samples were imaged by fluorescence microscopy, and the invasion rate was measured by 100 PVs. Scale bars, 50 μm. Results are presented as the mean ± S.D. from three independent experiments. ∗, *p* < 0.05 by Student’s *t* test.

GSK3β reversed the effects of prolonged hypoxia on HIF-1α protein levels, leading to increased HIF-1α stabilization and enhanced transcriptional activity.^25^ We aimed to investigate whether GSK3β is involved in the *C. atrati*-mediated anti-*T. gondii* response. Western blot analysis showed that phosphorylation of GSK3β at Ser9 was markedly reduced in a dose-dependent manner upon *C. atrati* treatment. Consistent with increased GSK3β activity, the phosphorylation level of β-catenin was elevated, whereas the protein levels of β-catenin, HIF-1α, and VEGF were reduced following *C. atrati* treatment (Figure 6A). The mRNA level of VEGF also decreased, whereas HIF-1α mRNA levels remained unchanged (Figure 6B). Interestingly, *T. gondii*-induced phosphorylation of GSK3β and its downstream target β-catenin, as well as the subsequent inhibition of protein degradation, were all reversed by *C. atrati*, suggesting that *C. atrati* suppresses *T. gondii* proliferation through GSK3β activation (Figure 6C).

To determine the role of GSK3β in *T. gondii* growth, we pre-treated *T. gondii*-infected ARPE-19 cells with the GSK3β inhibitor SB216763 and monitored *T. gondii* replication (Figure 6D). The results indicated that GSK3β inhibition enhanced *T. gondii* proliferation, increasing the proportion of PVs containing eight or even 16 tachyzoites. Moreover, SB216763 treatment also enhanced *T. gondii* invasion (Figure 6E), suggesting that GSK3β activity plays a crucial role in restricting *T. gondii* growth, possibly through its regulation of HIF-1α.

### 4′HAP suppressed *T. gondii* growth via GSK3β activity in ARPE-19 cells

**Figure 7:**
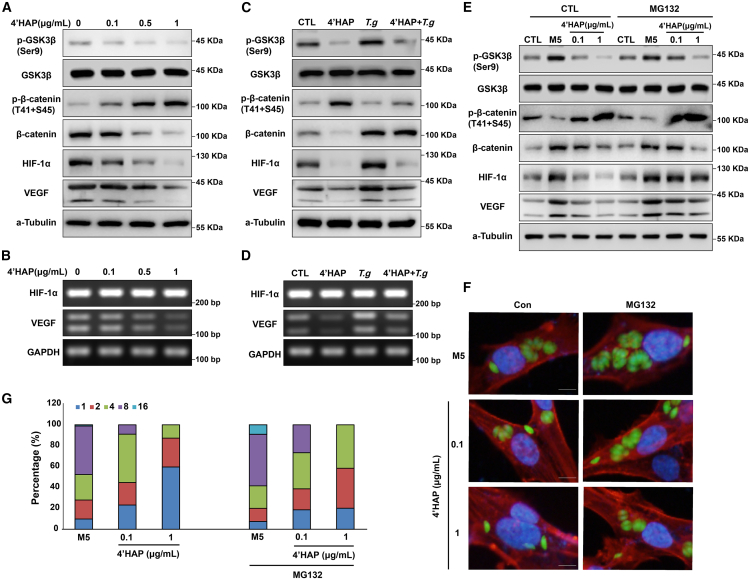
4′HAP represses the *T. gondii* growth by reducing HIF-1α stability via GSK3β/β-catenin pathway (A) Western blot analysis of phosphor- GSK3β/β-catenin, total- GSK3β/β-catenin, HIF-1α, and VEGF in ARPE-19 cells treated with 4′HAP for 24 h at the indicated concentrations. (B) mRNA levels of HIF-1α or VEGF measured by RT-PCR. ARPE-19 cells were pretreated with 4′HAP (10 μg/mL) and infected with *T. gondii* (MOI5) or left alone (CTL). (C) The protein levels of phosphor- GSK3β/β-catenin, total- GSK3β/β-catenin, HIF-1α, and VEGF were detected. (D) Measure the mRNA levels of HIF-1α and VEGF by RT-PCR. ARPE-19 cells were pretreated or untreated (CTL) with MG-132 for 4 h, and then treated with 4′HAP for 4 h at the indicated concentrations, and then infected with *T. gondii* for 24 h (E) The protein levels of phosphor- GSK3β/β-catenin, total- GSK3β/β-catenin, HIF-1α, and VEGF were measured by Western blot. (F) *T. gondii* proliferation was measured by confocal fluorescence. Scale bars, 10 μm. (G) The number of parasites per PV was counted and covered by percentage; a total of 100 PVs were counted. Results are presented as the mean ± S.D. from three independent experiments.

Next, we analyzed 4′HAP impact on GSK3β/HIF-1α signaling pathways modulated by *T. gondii* infection. Treatment with 4′HAP effectively reduced GSK3β phosphorylation at Ser9 while restoring phospho-β-catenin and total β-catenin levels to values similar to those in uninfected control cells (Figure 7A). Consistent with these changes in GSK3β activity, 4′HAP also affected downstream signaling pathways regulated by *T. gondii*. Specifically, *T. gondii*-induced elevation of HIF-1α protein levels and the upregulation of its target gene VEGF were significantly suppressed by 4′HAP treatment. VEGF mRNA levels also decreased, whereas HIF-1α mRNA levels remained unchanged (Figures 7A and 7B). Additionally, 4′HAP reversed *T. gondii*-induced changes in GSK3β/β-catenin phosphorylation, HIF-1α, and VEGF expression (Figures 7C and 7D).

To further investigate the role of the ubiquitin-proteasome system (UPS) in the regulation of intracellular HIF-1α levels by 4′HAP, we performed additional experiments using MG-132, a UPS inhibitor (Figure 7E). Consistent with the findings from *C. atrati* extract, MG-132 effectively prevented 4′HAP-induced degradation of HIF-1α, suggesting that 4′HAP regulates HIF-1α stability via the UPS. MG-132 supplementation restored *T. gondii* growth in the presence of 4′HAP (Figures 7F and 7G). These findings suggest that 4′HAP is the primary bioactive compound in *C. atrati* responsible for its anti-parasitic effects, likely through GSK3β-mediated regulation of HIF-1α stability.

### *In vivo* anti-parasitic activity of 4′HAP

**Figure 8:**
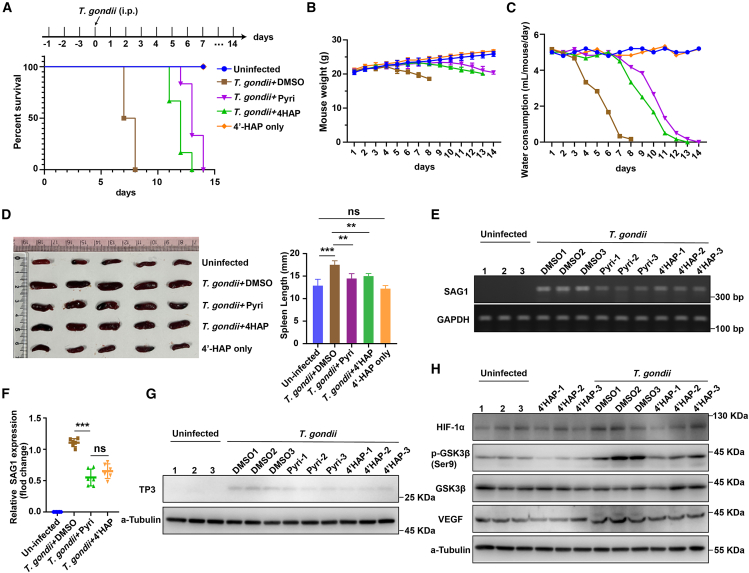
*In vivo* anti-parasitic activity of 4′HAP. 8-week-old male Mice were randomized to the indicated groups (*n* = 6 per group). Animals received intraperitoneal (i.p.) pretreatment with vehicle (DMSO), 4′HAP (1 mg/kg), or pyrimethamine (Pyri, 10 mg/kg) for three days, were then infected i.p. with *T. gondii* (2,000 tachyzoites per mouse), and subsequently continued on the same daily treatment (A) Kaplan-Meier survival curves; significance assessed by the log rank (Mantel-Cox) test. (B) Body weight was recorded daily. (C) Water consumption (mL/mouse/day) was recorded daily. (D) Representative spleen images and quantification of spleen length (*n* = 5). (E) Semi-quantitative RT-PCR of the *T. gondii* marker gene SAG1 from enucleated eyes (*n* = 3). (F) qPCR quantification of SAG1 expression (*n* = 6). (G) Western blot detection of the *T. gondii* protein TP3 in eyes (*n* = 3). (H) Western blot analysis of host signaling proteins (HIF-1α, *p*-GSK3β, total GSK3β, and VEGF) in the indicated groups in eyes (*n* = 3). Data are presented as mean ± SD from three independent experiments, each including one mouse per group (*n* = 3 biological replicates in total). For multi-grou*p* comparisons, one-way ANOVA with post-hoc multiple comparisons was used; ns, not significant; ∗∗, *p* < 0.01, ∗∗∗, *p* < 0.001.

To validate our *in vitro* findings, we evaluated the *in vivo* anti-parasitic activity of 4′HAP in male C57BL/6J mice intraperitoneally infected with *T. gondii* (2,000 tachyzoites per mouse). DMSO was used as the vehicle control, and pyrimethamine as the positive control. Mice received once-daily intraperitoneal dosing for three days before infection, which was continued daily thereafter. Kaplan-Meier analysis showed that 4′HAP significantly improved survival compared with infected vehicle controls, yielding a right-shifted curve comparable to pyrimethamine (Figure 8A). Consistent with this benefit, 4′HAP attenuated infection-associated weight loss and blunted the marked decline in water consumption observed in vehicle-treated mice (Figures 8B and 8C). Infection produced splenomegaly in the vehicle group, whereas 4′HAP significantly reduced spleen length toward the levels observed with pyrimethamine (Figure 8D).

At the molecular level, 4′HAP lowered tissue parasite burden. Semi-quantitative RT-PCR of ocular tissue showed diminished SAG1 signal after 4′HAP treatment (Figure 8E), which was confirmed by qPCR as a significant decrease in SAG1 transcript abundance (Figure 8F). Consistently, immunoblotting of eyes detected lower levels of the parasite protein TP3 in 4′HAP-treated mice (Figure 8G). We also found that 4′HAP decreased HIF-1α and its effector VEGF while normalizing GSK3β signaling, evidenced by reduced Ser9 phosphorylation with preserved total GSK3β (Figure 8H). These changes align with our *in vitro* data and suggest that 4′HAP dampens parasite-driven pseudo-hypoxic signaling to restrain *T. gondii* expansion. Collectively, 4′HAP demonstrates robust *in vivo* anti-parasitic efficacy comparable to pyrimethamine, supporting 4′HAP as a host-directed therapeutic candidate against *T. gondii*.

## Discussion

This study identifies *C. atrati* and its bioactive constituent, 4′HAP, as novel host-directed therapeutic agents against *T. gondii*. Our mechanistic investigation reveals that these compounds selectively inhibit the GSK3β/HIF-1α signaling axis, promoting ubiquitin-mediated degradation of HIF-1α and effectively counteracting the parasite’s exploitation of the host hypoxia adaptation mechanism (Figures 6, 7, and 8). These findings provide insights into a host-targeted strategy for controlling toxoplasmosis, broadening therapeutic options beyond conventional anti-parasitic treatments.

The apicomplexan parasite *T. gondii* causes toxoplasmosis, a ubiquitous and globally distributed parasitic disease that is generally asymptomatic but poses significant risks to fetuses and severely immunocompromised patients. Pyrimethamine and sulfadiazine, supplemented with folic acid, are the first-line drugs for treating toxoplasmosis. However, they cause severe side effects, and treatment failure occurs due to drug resistance.^26^^,^^27^^,^^28^ The lack of specific therapeutic agents and the limitations of existing drugs underscore the urgent need for novel, safe, and effective anti-*T. gondii* treatments. Natural compounds represent a promising source for discovering such therapies.

Our findings align with emerging evidence supporting the central role of HIF-1α in *T. gondii* pathogenesis. Studies have shown that *T. gondii* infection stabilizes HIF-1α by inhibiting its prolyl hydroxylation. This effect is mainly due to a significant reduction in the abundance of the key prolyl hydroxylase PHD2 during infection, which subsequently inhibits HIF-1α degradation.^16^ Furthermore, *T. gondii* induces HIF-1α activation via the activin-like kinase (ALK) receptor family, particularly ALK4. Overexpression of ALK4 enhances HIF-1 activity in *T. gondii*-infected cells in a kinase activity-dependent manner.^29^ GSK3β phosphorylates HIF-1α, promoting its interaction with the F box and WD repeat domain protein Fbw7, which mediates its ubiquitination and degradation.^18^ Additionally, *T. gondii*-secreted dense granule protein GRA18 interacts with regulatory elements of the host β-catenin destruction complex. By interacting with GSK3 and PP2A-B56, GRA18 increases β-catenin levels, thereby modulating host gene expression.^21^ These findings highlight the crucial role of the GSK3β/HIF-1α axis in supporting *T. gondii* survival within host cells. In this study, *C. atrati* and 4′HAP treatment significantly inhibited GSK3β activity, thereby promoting HIF-1α degradation via the ubiquitin-proteasome system. Pharmacological inhibition of GSK3β restored HIF-1α stability, thereby enhancing *T. gondii* replication and survival, further confirming the essential role of this pathway. Moreover, VEGF, a key transcriptional target of HIF-1α,^30^ was significantly downregulated, effectively impairing the pro-survival signaling pathways exploited by *T. gondii*. Importantly, we observed concordant *in vivo* effects in an intraperitoneal infection model: 4′HAP improved survival, mitigated infection-associated weight loss and water-intake decline, reduced splenomegaly, and lowered ocular parasite burden, while likewise decreasing HIF-1α/VEGF and normalizing GSK3β signaling (Figure 8). These findings emphasize the broader impact of *C. atrati* and 4′HAP on mitigating hypoxia-induced signaling during infection. By targeting the GSK3β/HIF-1α axis, this study presents a potential strategy for managing other intracellular infections, including tuberculosis and chronic viral diseases, where hypoxia signaling is critical. These findings open new avenues for combining host-targeted and traditional therapies to improve treatment outcomes.

### Limitations of the study

This work provides *in vivo* proof of concept for *C. atrati* and 4′HAP, but several limitations remain. First, efficacy was tested in a single acute intraperitoneal model, with one dosing schedule and a single sex/age cohort; broader validation across strains (including cyst-forming), infection routes (oral), therapeutic regimens, and both sexes is needed. Second, the study was not powered for formal non-inferiority versus pyrimethamine, and comprehensive blinding, pre-specified stopping rules, and toxicity readouts were not implemented. Third, pharmacokinetics/pharmacodynamics (bioavailability, exposure-response, and ocular/brain distribution) and standard toxicology were not assessed; formulation beyond DMSO was not optimized. Mechanistic evidence is correlative (signaling changes ex *vivo*); genetic or pharmacologic rescue and direct target-engagement assays are required. Finally, the extract batch standardization and potential drug-drug interactions were not examined. Future studies should focus on following directions: (i) PK/PD and formulation optimization; (ii) efficacy across genotypes, routes, and therapeutic timing with powered, blinded endpoints; (iii) immunoprofiling of innate/adaptive responses; (iv) mechanistic validation of the GSK3β/HIF-1α axis (genetic perturbation, binding/chemoproteomics); and (v) combination studies with frontline anti-parasytics to assess additivity/synergy and safety.

## Resource availability

### Lead contact

Further information and requests for resources and reagents should be directed to and will be fulfilled by the lead contact, Guang-Ho Cha (gcha@cnu.ac.kr).

### Materials availability

This study did not generate unique reagents.

### Data and code availability

The target genes of 4′HAP in this paper were obtained from CMSP (https://tcmsp-e.com/tcmsp.php), SwissTargetPrediction (http://www.swisstargetprediction.ch/), Charité Prediction platform (https://bioinformatics.charite.de/main/index.php), and PubMed databases (https://pubmed.ncbi.nlm.nih.gov/).

Original western blot images have been deposited at Mendeley at [https://doi.org/10.17632/84395nrz42.1] and are publicly available as of the date of publication. Microscopy data reported in this paper will be shared by the lead contact upon request.

This paper does not report original code.

Any additional information required to reanalyze the data reported in this paper is available from the lead contact upon request.

## STAR★Methods

### Key resources table

**Table:** 

REAGENT or RESOURCE	SOURCE	IDENTIFIER
**Antibodies**		

GFP (B2)	Santa Cruz Biotechnology	Cat#sc-9996; RRID: AB_627695
TP3	Santa Cruz Biotechnology	Cat#sc-52255; RRID: AB_630350
α-Tubulin	Santa Cruz Biotechnology	Cat#sc-32293; RRID: AB_628412
HIF-1α	Cell Signaling Technology	Cat#3716S; RRID: AB_2116962
Phospho-GSK-3β (Ser9)	Cell Signaling Technology	Cat#9323SS; RRID: AB_2115201
GSK-3β	Cell Signaling Technology	Cat#3716S; RRID: AB_490890
Anti-β-catenin (phosphor T41 + S45)	Abcam	Cat#ab81305; RRID: AB_1640288
Anti-β-catenin non-phospho (active) S45 antibody	Abcam	Cat#ab305261
Anti-VEGF	iReal Biotechnology	Cat#IR108-442
Anti-rabbit-horseradish peroxidase	Immuno Research Laboratories	Cat#323-005-024; RRID: AB_2315781
Anti-mouse-horseradish peroxidase	Immuno Research Laboratories	Cat#223-005-024; RRID: AB_2339261
Goat anti-Mouse IgG (H + L) Cross-Adsorbed Secondary Antibody, Alexa Fluor™ 568	Thermo Fisher Scientific	Cat#A-11004; RRID: AB_2534072

**Chemicals, peptides, and recombinant proteins**		

4′-Hydroxyacetophenone	Sigma-Aldrich	Cat#278564
*Cynanchi atrati Radix*	provided by Dr. Chang Gue Son from the Daejeon Regional Cancer Center	–
Azelaic acid	Sigma-Aldrich	Cat#246379
Suberic acid	Sigma-Aldrich	Cat#S5200
Pyrimethamine	Sigma-Aldrich	Cat#SML3579
2′-Hydroxy-5′-methoxyacetophenone	Tokyo Chemical Industry	Cat#H0868
2′-Hydroxy-4′-methoxyacetophenone	Tokyo Chemical Industry	Cat#H0789
Oleanolic acid hydrate	Selleckchem	Cat#S2334
Syringic acid	Selleckchem	Cat#S3629
2′,4′-Dihydroxyacetophenone	MedChemExpress	Cat#HY-Y0694
Sibiricose A1	MedChemExpress	Cat#HY-N8208
SB216763	MedChemExpress	Cat#HY-12012
4′-Hydroxy-3′-methoxyacetophenone	Thermo Fisher Scientific	Cat#A1043918
Lipofectamine™ RNAiMAX	Thermo Fisher Scientific	Cat#13778075

**Critical commercial assays**		

CellTiter 96 AQueous MTS Reagent	Promega	Cat#G1111
M-MLV reverse transcriptase kit	Thermo Fisher Scientific	Cat#28025013

**Deposited data**		

Original western blot images and RT-PCR gel images	Mendeley Data	https://doi.org/10.17632/84395nrz42.1

**Experimental models: Cell lines**		

ARPE-19 cells	ATCC	CRL-2302
HFF-1 cells	ATCC	SCRC-1041

**Experimental models: Organisms/strains**		

C57BL/6J mouse BomTac	Damool Science (Daejeon, Republic of Korea)	RRID: IMSR_TAC: B6JBOM
*Toxoplasma gondii* RH expressing transgenic green fluorescent protein	provided by Dr. Yoshifumi Nishikawa (Obihiro University of Agriculture and Veterinary Medicine)	–
*Toxoplasma gondii* RH	provided by Dr. Yoshifumi Nishikawa (Obihiro University of Agriculture and Veterinary Medicine)	–

**Oligonucleotides**		

See Table S1 for oligonucleotides used in this paper	This paper	–
HIF-1α siRNA	Santa Cruz Biotechnology	Cat#sc-35561

**Software and algorithms**		

ImageJ software	National Institutes of Health	https://imagej.net/ij/
R software	R Core Team (2024)	https://www.R-project.org/
GraphPad Prism 8.0.1	GraphPad Software	https://www.graphpad.com
FlowJo v10	BD Biosciences	https://www.flowjo.com
PubChem database	NCBI	https://pubchem.ncbi.nlm.nih.gov/
PyMOL 2.3.4 software	Schrödinger, LLC	https://www.pymol.org/
Open Babel 2.3.2 software	Open Babel Project	https://openbabel.org/
AutoDock Vina 1.1.2	Scripps Research Institute	https://autodock-vina.readthedocs.io/
TCMSP (Traditional Chinese Medicine Systems Pharmacology)	Northwest A&F University (NWSUAF) & collaborators	https://tcmsp-e.com/tcmsp.php
SwissTargetPrediction	University of Lausanne & SIB Swiss Institute of Bioinformatics	https://www.swisstargetprediction.ch/
Charité target-prediction platform	Charité – Universitätsmedizin Berlin	https://bioinformatics.charite.de/main/index.php
PubMed database	NCBI	https://pubmed.ncbi.nlm.nih.gov/

### Experimental model and study participant details

#### Animals

All animal procedures were approved by the Institutional Animal Care and Use Committee (IACUC) of Chungnam National University (approval no. 202412A-CNU-237; Daejeon, Republic of Korea) and complied with the National Institutes of Health guidelines. Male C57BL/6J mice (8 weeks old; Damool Science, Daejeon, Korea) were housed five per sawdust-bedded cage under a 12-h light/dark cycle with *ad libitum* access to standard chow and water. Mice were randomized (*n* = 6 per group) into four groups: uninfected blank control, infected + vehicle (DMSO), infected +4′HAP (1 mg/kg/day),^31^ and infected + pyrimethamine (10 mg/kg/day).^32^ Treatments were administered once daily by intraperitoneal (i.p.) injection for 3 days before infection and continued once daily thereafter. Infection was performed i.p. with *T. gondii* tachyzoites (2,000 per mouse in 150 μL sterile buffer). Survival was recorded daily; body weight and water intake (mL per mouse per day; cage-level volume normalized per mouse) were measured once daily. At experimental endpoints, spleens were photographed with a scale bar and length quantified in ImageJ; eyes were collected for RT-PCR/qPCR and immunoblotting.

#### Cell culture

The human retinal pigment epithelial cell line ARPE-19, which consists of highly polarized cells from the outer blood-retina barrier between the photoreceptors of the neurosensory retina and vascularized choroid, was obtained from the American Type Culture Collection (ATCC). Cells were routinely cultured under 5% CO_2_ at 37°C in DMEM/F12 medium (WelGENE) supplemented with 10% fetal bovine serum (FBS, Gibco BRL) and 1% antibiotic-antimycotic reagents (Gibco BRL). The cells were passaged with 0.25% Trypsin-EDTA (Life Technologies) every 3 days. ARPE-19 cells between passages 4 and 8 were used in this study.

Human Foreskin Fibroblast-1 (HFF-1 cells, ATCC, SCRC-1041) were included as a second host cell model and were maintained under the same conditions as ARPE-19 (DMEM/F12 with 10% FBS and 1% antibiotic–antimycotic at 37 °C, 5% CO_2_; passaged with 0.25% trypsin-EDTA every 3 days). Both cell lines were confirmed by PCR to be mycoplasma-free prior to experimentation, and no contamination was detected during the study period.

#### Parasites

Toxoplasma gondii tachyzoites RH strain was maintained in ARPE-19 cells at 37°C, 5% CO2 and passaged for every 2–3 days. RH expressing transgenic green fluorescent protein (GFP-RH) was kindly provided by Dr. Yoshifumi Nishikawa (Obihiro University of Agriculture and Veterinary Medicine) and incubated same condition with RH.

### Method details

#### Cytotoxicity

ARPE-19 cells were seeded into 96-well plates at a density of 0.5 × 10^4^ cells/mL and cultured at 37°C under 5% CO_2_ conditions. After 24 h, when the cells reached approximately 80% confluence, they were treated with different concentrations of *C. atrati* (which was generously provided by Dr. Chang Gue Son from the Daejeon Regional Cancer Center and prepared exactly as described in Son et al.^33^) or 4′HAP, maintaining the same volume across treatments. Each concentration was prepared in triplicate. After 24 h of treatment, 20 μL of MTS reagent was added to each well, and the plates were incubated for 1 h at 37°C under 5% CO_2_ in the dark. Absorbance was measured at 490 nm using a microplate reader, and the average values were calculated.

#### Analysis of intracellular *T. gondii* proliferation

Quantification of intracellular replication by counting tachyzoites per parasitophorous vacuole (PV) was performed as previously described,^34^ with minor modifications as detailed below. ARPE-19 cells were cultured in 24-well plates containing glass coverslips and serum-starved 4h prior to infection with GFP-RH at an MOI5. One hour post-infection, cells were treated with *C. atrati* extract or commercially sourced reference compounds reported from *C. atrati* (see Reagents, 1 μg/mL) and incubated for an additional 24 h. The coverslips were washed with PBS and then fixed with 4%formaldehyde. The cells were stained with Texas Red-X phalloidin (Life Technologies Corporation, CA, USA) to label F-actin and mounted on slides using mounting medium with DAPI (Vector Laboratories, Burlingame, CA, USA). The cells were then imaged using fluorescence microscopy or confocal microscopy. One to two hundred parasitophorous vacuoles (PVs) were randomly selected in each preparation, and parasite replication was monitored by counting the number of tachyzoites per PV, and PVs were classified by division state (e.g., 2, 4, 8, 16 tachyzoites per PV). Where noted, total PV number per field was also recorded to distinguish invasion frequency (PV formation) from replication per PV. Three separate experiments were performed for statistical analysis of the results.

#### Analysis of intracellular *T. gondii* growth by flow cytometry

Flow-cytometric quantification of intracellular *T. gondii* burden was performed as previously described,^35^ with minor modifications as detailed below. ARPE-19 cells were seeded in 12-well plates and serum-starved for 4 h prior to treatment with *C. atrati* or commercially sourced reference compounds reported from *C. atrati* (see Reagents, 1 μg/mL). Then the cells were infected with GFP-RH at MOI5. After 24h incubation, the cells were washed with PBS and added 0.25%Trypsin-EDTA to detach then neutralized by 10% FBS DMEM media. The collected cells were washed by FACS buffer (1% BSA in PBS), then analyzed on a FACScan (BD Bio-science).

#### Sample preparation to monitor the *T. gondii* infection on host cell or reagent effect on *T. gondii* growth

To compare host-protein changes between uninfected ARPE-19 cells and infected cells and to evaluate reagent effects on *T. gondii* growth, ARPE-19 cells were serum-starved for 4 h, then refreshed with new medium. For anti-parasitic testing, cells were pre-treated for 4 h with the indicated reagent (*C. atrati* extract or 4′HAP), followed by infection with *T. gondii* at an MOI of 5. Reagents were maintained during the 24 h infection period (co-treatment). At the endpoint, cells from each group were harvested and processed for the planned analyses as described in the corresponding sections.

#### Western blot analysis

Proteins were extracted from ARPE-19 cells treated as described in Section 4′HAP suppressed T. gondii growth via GSK3β activity in ARPE-19 cells with PRO-PREP Protein Extraction Solution (iNtRON Biotechnology) and then splitted for 30 min on ice, followed by boiling for 10 min. The same amount of proteins was loaded into the SDS-PAGE gel and separated by electrophoresis. Then, proteins were transferred to polyvinylidene difluoride (PVDF) membrane (Bio-Rad Laboratories). The membranes were blocked with 5% skim milk in Tris-buffered saline, including 0.1% Tween 20 (TBST) for 1 h at room temperature. After being washed twice in TBST for 5 min, membranes were incubated with indicated primary antibodies (1:1000 diluted in 5% BSA supplemented with TBST) overnight at 4°C. Following washed three times with TBST, membranes were incubated with HRP-conjugated anti-rabbit or anti-mouse secondary antibody (1:5000 diluted in 5% skim milk supplemented with TBST) for 1 h at room temperature. After three washes, the membranes were soaked with Immobilon Western Chemiluminescent HRP Substrate (Merck Millipore), and chemiluminescence was detected using a Fusion Solo System (Vilber Lourmat).

#### RNA isolation and RT-PCR

Total RNA was isolated from ARPE-19 cells processed under the same infection and treatment conditions described in Section 4′HAP suppressed T. gondii growth via GSK3β activity in ARPE-19 cells using the Trizol reagent (Invitrogen), and cDNA was generated using M-MLV reverse transcriptase kit (Invitrogen Life Technologies) as described by the manufacturer. All PCR reactions were performed with a MyCycler (Bio-Rad) for 35 cycles. The primers were designed using Primer 3 software. mRNA sequence of the selected genes was obtained from NCBI website. The primer sequences are summarized in Table S1. Amplified products were electrophoresed in a 1.5% agarose gel and visualized with ethidium bromide. Quantification of mRNA was performed using an imaging densitometer (Bio-Rad Laboratories, Inc).

#### Molecular docking

The chemical structure file of the 4′-hydroxyacetophenone was obtained from the PubChem database (https://pubchem.ncbi.nlm.nih.gov/#query=4%E2%80%99-Hydroxyacetophenone) and converted from SDF to PDB format using Open Babel 2.3.2 software. The GSK3β (PDBID:1J1B) structure was retrieved from the Protein DataBank (PDB). Water molecules and ligands were removed from the receptor structure using PyMOL 2.3.4 software. The receptor protein was subsequently prepared using AutoDockTools by adding hydrogens and assigning charges, and both the receptor and ligand molecules were converted to pdbqt format. Molecular docking was performed using AutoDock Vina 1.1.2. The docking results were analyzed with Discovery Studio, and the binding modes were visualized using PyMOL.

#### Computational prediction of 4′HAP targets followed by Hallmark, KEGG, and GO enrichment

Putative targets of 4′-hydroxyacetophenone were collected from TCMSP (https://tcmsp-e.com/tcmsp.php), SwissTargetPrediction (https://www.swisstargetprediction.ch/), the Charité target-prediction platform (https://bioinformatics.charite.de/main/index.php), and PubMed (https://pubmed.ncbi.nlm.nih.gov/). After merging and removing duplicates by HGNC gene symbol, 188 unique human targets remained from 194 initial records. Identifier harmonization used UniProt/biomaRt with obsolete symbols pruned; the statistical background (universe) was set to all human protein-coding genes available in the respective annotation.

Functional enrichment analyses were performed in R using clusterProfiler and msigdbr. Hallmark over-representation analysis (hypergeometric test) was conducted against the MSigDB Hallmark collection (Homo sapiens); KEGG pathway enrichment used enrichKEGG (organism “hsa”); Gene Ontology enrichment used enrichGO with org. Hs.e.g.,.db annotation, analyzing BP/CC/MF separately. *p* values were adjusted by Benjamini–Hochberg, and terms with FDR <0.05 were considered significant. Where applicable, redundant GO terms were summarized with simplify (similarity cutoff 0.7). Visualization (dot/bar plots and ranked summaries) was generated with clusterProfiler/ggplot2.

#### Transfection

One day before transfection, ARPE-19 cells were cultured in a 12-well plate with glass coverslips. Diluting siRNA (siHIF-1α) duplex in 125 μL Opti-MEM to make final concentration of 100 nM. Lipofectamine RNAiMAX (Life Technologies Corporation) was gently mixed with 125 μL media and incubated for 5 min at room temperature. After that mixed them together and incubated for 20 min at room temperature. The mixture was added to each well-containing cells. The final volume in each well is 1 mL. After incubating 24 h at 37°C, the media were changed to starvation medium for 4 h and infected with GFP-RH, the samples were collected after 24 h, and parasite division time was analyzed by fluorescent microscopy.

### Quantification and statistical analysis

All experiments were repeated at least three times, independently, and the results were expressed as the mean ± standard deviation (SD). *p* values between groups were determined by a two-tailed paired Student’s *t* test, and three or more groups were analyzed by one-way ANOVA followed by post-hoc multiple comparisons. Data were analyzed using GraphPad Prism software. *p* < 0.05 was considered “statistically significant. Statistical details can be found in the figure legends.

## Acknowledgments

This work was supported by the Basic Science Research Program of the 10.13039/501100003725National Research Foundation of Korea (NRF) funded by the 10.13039/501100004085Ministry of Education, Science and Technology (2018R1D1A1B07050779), (2021R1I1A2055834), and the National Research Foundation (NRF) of Korea grant funded by the Korea government (MSIT) (RS-2025-16068439) (CGH). This work was also supported by the Natural Science Foundation of Guangdong province (2023A1515012483) and the Competitive Allocation Project of Zhanjiang Municipal Science and Technology Development Special Fund (2022A01156) (GFF). Additionally, this work was supported by BK21 FOUR Program by Chungnam National University Research Grant, 2025 (HGH), and by 10.13039/501100002462Chungnam National University (HGM). The funders had no role in study design, data collection and analysis, publication decision, or manuscript preparation.

## Author contributions

F.-F.G.: conceptualization, methodology, data curation, and investigation; G.-H.H.: writing – original draft, formal analysis, investigation, and visualization; X.-C.W.: investigation, software, and formal analysis; Y.-S.Y. and J.-h.Z.: data curation, validation, and software; W.Z. and I.-W.C.: formal analysis and visualization; J.-M.Y.: validation and resources; X.-t.C.: supervision and conceptualization; G.M.H.: supervision, methodology, and writing – review & editing; G.-H.C.: conceptualization, writing – review & editing, funding acquisition, supervision, and project administration.

## Declaration of interests

No potential conflicts of interest were disclosed.
