# Supplementary material for: GSK3β/HIF-1α signaling-dependent anti-parasite effect of Cynanchi atrati Radix
Source: iScience. 2025 Dec 1;29(1):114292. doi: 10.1016/j.isci.2025.114292 (PMC12774676; doi:10.1016/j.isci.2025.114292)
Supplement: Document S1. Figures S1–S4 and Table S1 [file mmc1.pdf]

iScience, Volume *29*

Supplemental information

GSK3β/HIF-1α signaling-dependent anti-parasite effect of *Cynanchi atrati Radix*

Fei-Fei Gao, Guan-Hao Hong, Xin-Cheng Wang, Jia-hui Zeng, Yu-Sun Yun, In-Wook Choi, Jae-Min Yuk, Wei Zhou, Xin-tian Chen, Gang Min Hur, and Guang-Ho Cha

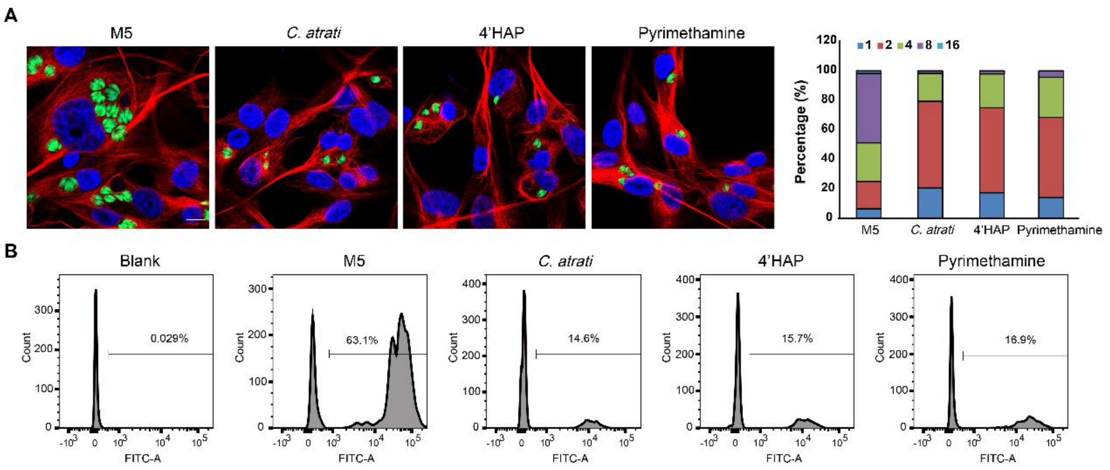

Supplemental Figure S1. Comparative anti-*T. gondii* activity of *C. atrati* and 4′HAP in ARPE-19 cells, with pyrimethamine as a positive control. Related to Figure 1. ARPE-19 cells infected with *T. gondii* (MOI 5), and then treated with *C. atrati* (1 μg/mL), 4′HAP (1 μg/mL), or pyrimethamine (positive control, 10 μg/mL) for 24h (A) Representative fluorescence micrographs showing intracellular parasite proliferation. Scale bar, 10 μm. (B) ARPE-19 cells were pretreated with *C. atrati* (1 μg/mL), 4′HAP (1 μg/mL), or pyrimethamine (positive control, 10 μg/mL) for 4h and then infected with *T. gondii* (MOI 5) for 24 h. Median fluorescence intensity (MFI) was detected by FACS. Results are presented as the mean ± S.D. from 3 independent experiments.

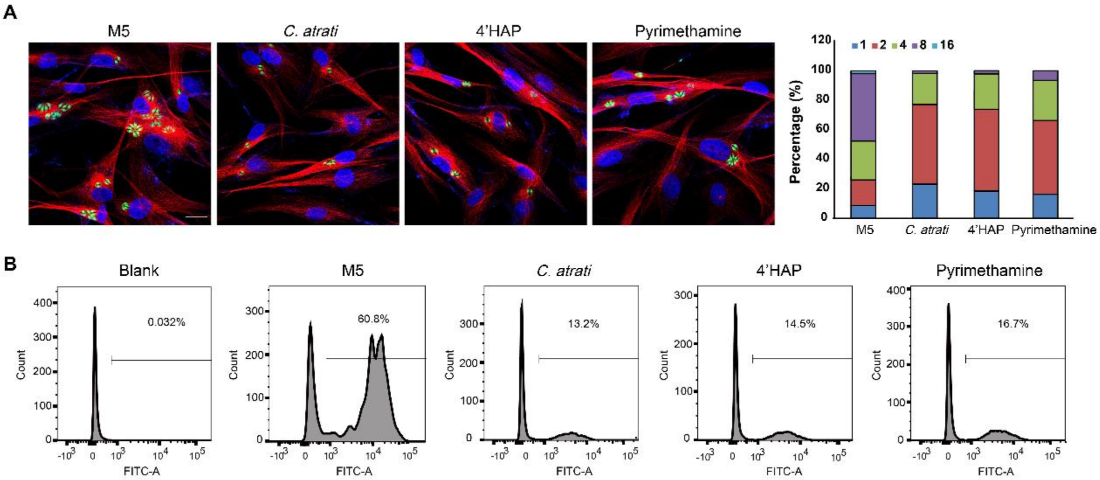

Supplemental Figure S2. Anti-*T. gondii* efficacy of *C. atrati* and 4′HAP in HFF-1 cells. Related to Figure 1. HFF-1 cells infected with *T. gondii* (MOI 5), and then treated with *C. atrati* (1 μg/mL), 4′HAP (1 μg/mL), or pyrimethamine (positive control, 10 μg/mL) for 24h (A) Representative fluorescence micrographs showing intracellular parasite proliferation. Scale bar, 20 μm. (B) HFF-1 were pretreated with *C. atrati* (1 μg/mL), 4′HAP (1 μg/mL), or pyrimethamine (positive control, 10 μg/mL) for 4h and then infected with *T. gondii* (MOI 5) for 24 h. Median fluorescence intensity (MFI) was detected by FACS. Results are presented as the mean ± S.D. from 3 independent experiments.

A

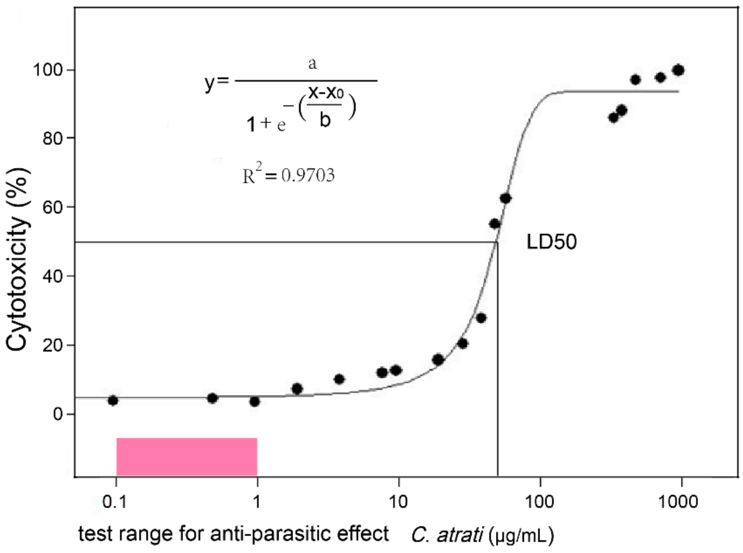

Cytotoxocity of *C. atrati* in ARPE-19 cells

B

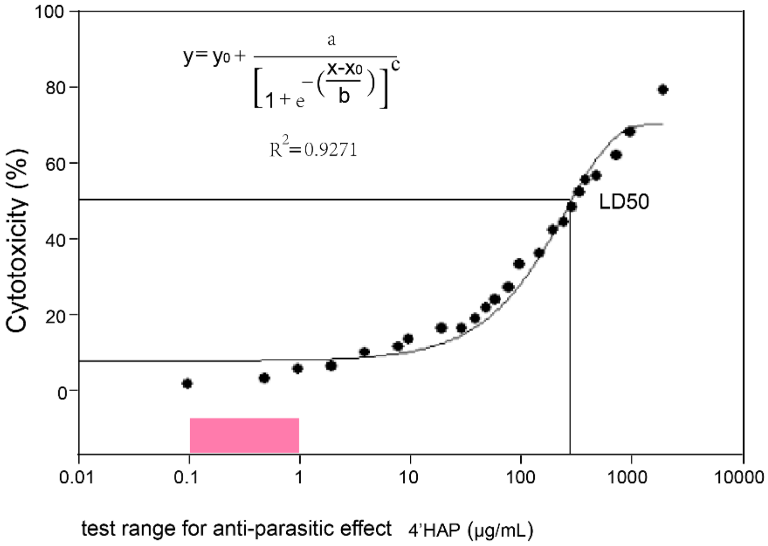

Cytotoxocity of 4'HAP in ARPE-19 cells

Supplemental Figure S3. Cytotoxicity of *C. atrati* and 4'HAP in ARPE-19 cells measured by the MTT assay. Related to Figure 1. A/B. ARPE-19 cells were treated with increasing concentrations of *C. atrati* or 4'HAP for 24 h, and cell viability was assessed using the MTT assay. The half-maximal lethal dose (LD50) is indicated on the graph. Each data point represents the mean ± (SD) of 3 independent experiments performed in triplicate.

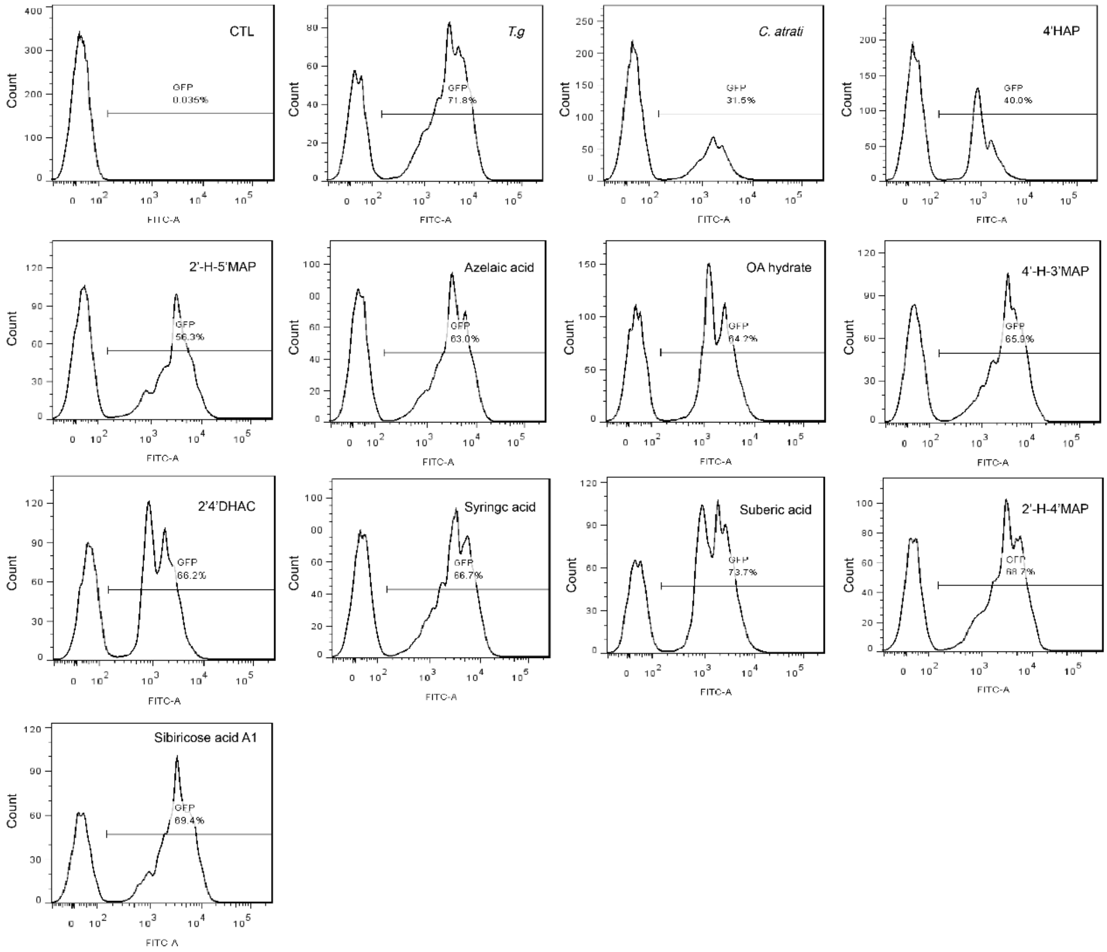

Supplemental Figure S4. Screening of commercially sourced reference compounds reported from *C. atrati* for inhibition of *T. gondii* proliferation by FACS. Related to Figure 2. ARPE-19 cells were pretreated with commercially sourced reference compounds reported from *C. atrati* (1 μg/mL) for 4 h and infected with *T. gondii* (MOI5) for 24h. Median fluorescence intensity (MFI) was detected by FACS. Results are presented as the mean ± S.D.

Table 1 Primer sequences used for RT-PCR in this study.

Name	Forward 5′→3′	Reverse 5′→3′
h-*HIF1A*	TGAGCTTGCTCATCAGTTGC	CCAGAAGTTTCCTCACACGC
h-*VEGFA*	TTGCCTTGCTGCTCTACCTCCA	GATGGCAGTAGCTGCGCTGATA
SAG1	GCTGTAACATTGAGCTCCTTGATTCCTG	CCGGAACAGTACTGATTGTTGTCTTGAG
h-*GAPDH*	GTCTCCTCTGACTTCAACAGCG	ACCACCCTGTTGCTGTAGCCAA
m-*Gapdh*	CCCAGCTTAGGTTCATCAGGTA	CAACAATCTCCACTTTGCCACT
